# Work-focused healthcare from the perspective of employees living with cardiovascular disease: a patient experience journey mapping study

**DOI:** 10.1186/s12889-023-16486-x

**Published:** 2023-09-11

**Authors:** Marije E. Hagendijk, Nina Zipfel, Floor J. Oomen, Jan L. Hoving, Philip J. van der Wees, Carel T. J. Hulshof, Ersen B. Çölkesen, Marijke Melles, Sylvia J. van der Burg-Vermeulen

**Affiliations:** 1 Department of Public and Occupational Health, Coronel Institute of Occupational Health, Amsterdam Public Health Research Institute, Amsterdam UMC Location University of Amsterdam, Amsterdam, The Netherlands; 2 Faculty of Industrial Design Engineering, Delft University of Technology, Delft, The Netherlands; 3 Scientific Institute for Quality of Healthcare (IQ Healthcare), Radboud University Medical Centre, Nijmegen, The Netherlands; 4 Department of Cardiology, St. Antonius Hospital, Nieuwegein, The Netherlands

**Keywords:** Sick leave, Occupational Health, Cardiology, Occupational health services, Qualitative research

## Abstract

**Background:**

People living with cardiovascular diseases (CVD) often experience work participation problems. Good work-focused healthcare, defined as the received advice, treatment, and guidance focusing on work participation, can support the patient and work place. However, experiences with work-focused healthcare are generally not always positive which is a barrier for work participation. Therefore, the objective of this study is to gain insight into the work-focused healthcare journey from the perspective of patients with work participation problems due to CVD, to understand their experiences and needs, and to derive opportunities for improving work-focused healthcare service at a system level.

**Methods:**

Semi-structured interviews, preceded by preparatory assignments, were conducted with 17 patients who experience(d) work participation problems due to CVD. The patient experience journey map (PEJM) approach was used to visualize the patients’ work-focused healthcare journey, including experiences and needs over time and place, from which opportunities to improve work-focused healthcare from the patient’s perspective were derived.

**Results:**

An aggregated PEJM consisting of six phases was composed and graphically mapped. The first phase, *working*, represents a period in which CVD health problems and subsequent functional limitations occur. The next two phases, *short-* and *long-term sick leave*, represent a period of full sick leave. The last three phases*, start-, partial-, and full vocational reintegration*, focus on the process of return to work that takes place ranging from a few months up to several years after sick-listing. For each phase the touchpoints, timespan, stakeholders, activities, experiences and needs from the perspective of the patients were identified. Finally, for better work-focused healthcare nine opportunities for improvement were derived from the PEJM, e.g. emphasize the need for work adjustment prior to the medical intervention, provide more personalized advice on handling work limitations, and putting more compelling pressure on the employer to create suitable work positions for their employees.

**Discussion/conclusion:**

This paper contributes insights to provide a more patient-centered work-focused healthcare trajectory for patients employed in paid jobs when living with CVD. The PEJM provides an understanding of the patients’ perspectives throughout their work-focused healthcare journey and highlights opportunities for improvement towards a better suited and seamless patient journey, Although this research was conducted within the Dutch healthcare system, it can be assumed that the findings on integrated work-focused healthcare are largly transferable to other healthcare systems.

**Supplementary Information:**

The online version contains supplementary material available at 10.1186/s12889-023-16486-x.

## Background

Cardiovascular diseases (CVD) are the leading cause of chronic disease morbidity and mortality industrialized countries [1, 2]. A large proportion of these individuals already live with CVD during the working age [3]. This number is expected to increase further due to the rising retirement ages in Western countries, such as the Netherlands [4–6]. Within the working population, CVD often leads to temporary or prolonged (partial) sick leave [7, 8], and factors such as mental health problems and negative perceptions are reported as barriers to successful return to work (RTW) [9–11]. However, work participation is crucial for improving health and wellbeing [12]. Therefore, CVD patients with disease-related presenteeism and sickness absence express the need for work-focused healthcare that supports staying at work, or returning to work, thus helping them overcome these barriers [13, 14]. Work-focused healthcare refers to the advice, treatment, guidance and support received, with a specific focusing on work participation [15]. Professionals providing work-focused healthcare can include those from occupational healthcare, such as occupational physicians and labor experts, as well as clinical care professionals like cardiologists and cardiac rehabilitation specialists [16–18].

However, previous studies have indicated that people living with CVD did not always have positive experiences with the current work-focused healthcare system. They reported a lack of (early) advice regarding RTW, a lack of motivation to RTW from professionals and a lack of follow-up appointments to discuss RTW [13, 14, 19]. These negative experiences were attributed to a lack of knowledge and awareness about the topic work of healthcare professionals [20], and the use of a one-size fits all approach in work-focused healthcare systems [21]. These negative experiences were identified as barriers to work participation. Therefore, earlier literature suggested to need to better align the organization of the work-focused healthcare delivery system with the needs and preferences of patients, known as patient-centered care [21–23].

To implement true patient-centered care within the organization of work-focused healthcare for patients with CVD, it is essential to thoroughly understand how these patients experience work-focused healthcare services and their related needs over time (e.g. short-term and long-term sick leave) and in different settings (e.g. at home, at work, during consultations). This understanding should also take into account the specific factors of the healthcare system being mapped [24]. Gaining insights into patients’ positive and negative experiences, and how they relate to their needs requires comprehending healthcare services at a system level [25]. Patient experience journey mapping (PEJM), an approach from the field of human-centred design, is a method that enables an aggregated graphical representation of sociotechnical healthcare services at a system level capturing patients’ experiences and needs over time and in different settings [25–27]. The PEJM approach thus facilitates the identification of opportunities to improve the healthcare service to better meet patients’ needs [26].

The objective of this study is to gain insights into the work-focused healthcare journey from the perspective of patients with work participation problems due to a CVD, to understand their experiences and needs, and to derive opportunities from these experiences and needs for improving work-focused healthcare service at a system level. The research questions are as follows: (1) What does the work-focused healthcare journey look like for patients who experience work participation problems due to a CVD? (2) What are the experiences and needs of these patients during their work-focused healthcare journey? (3) Which opportunities for improvement can be derived from the patients’ experiences and needs regarding work-focused healthcare over time and in different settings? Since this study is conducted in the context of the Dutch healthcare system, an explanation of the work-focused healthcare system in the Netherlands can be found in text Table 1.

**Table 1:** Work-focused healthcare for employees in the Netherlands

Different from other healthcare systems worldwide, in the Dutch work-focused healthcare system there is a strict division between the medical roles of clinical and occupational healthcare professionals [28]. Clinical healthcare professionals are involved in treating the patients’ disease, while occupational healthcare professionals are responsible for certifying sickness absence, providing return to work guidance, and assessing social security benefits, as regulated by the Dutch Improved Gatekeeper Act and the Act on Work and Income according to Work Capacity. This strict division between the clinical and occupational roles is for occupational healthcare professionals to perform their tasks, as providing sick notes, without any conflict of interest by a physician–patient relationship [29].
Work-focused healthcare for employees is mainly delivered by occupational healthcare professionals, including occupational physicians, insurance physicians and labor experts. When an employee reports sick to their employer, the employer is financially responsible for the first two years [30]. Additionally, the employer has a legal obligation to contract an occupational health service and an occupational physician within the first week of the employee’s sick-leave [30]. The occupational physician must provide a problem analysis and return to work plan for sick employees six weeks after the start of the sick leave. Every employee has the legal right to consult an occupational physician [31]. In current practice, occupational healthcare is often delivered by case managers and occupational health nurses under delegated responsibility of an occupational physician [32].
After two years, an insurance physician working for the Dutch Social Security Institute: the Institute for Employee Benefit Schemes (SSA) assesses whether the sick-listed employee is eligible for a long-term disability benefit [33]. The Sickness Benefits Act provides for workers who are sick-listed and no longer have an employment contract. After reporting sick, these workers receive sickness benefit and are entitled to occupational healthcare by the SSA during the sickness benefit period.

## Methods

### Design and setting

To gain insight into the work-focused healthcare system from the perspective of patients living with CVD and to identify potential areas for improvement throughout the care system, we conducted a qualitative data collection using semi-structured interviews. The collected data was then analysed using the PEJM approach, which is a method derived from the human-centered design discipline. This approach aims to analyse patients’ experiences within the sociotechnical system studied and to identify areas where improvements can be made to enhance the overall experience of patients [26]. The Consolidated criteria for reporting qualitative research (COREQ) checklist was used for reporting the methods and results [34].

### Participants

#### Inclusion criteria

Individuals were eligible to participate if they met the following criteria: 1) diagnosed with and having received healthcare for CVD; 2) of working age (between 18 and 67 years); 3) employed in paid work (temporary or permanent employment contract) at the moment of CVD diagnosis; 4) experienced work participation problems due to CVD that resulted in (partial) sick leave or adjustments in work for at least six weeks as this aligns with the point when occupational health consultation starts within the Dutch work-focused healthcare system; and 5) fluently speak and understand the Dutch language.

#### Recruitment of the participants

Participants were obtained from two different sources to ensure a variety of time points and locations within the (work-focused) healthcare system. First, participants were recruited through purposive sampling by a personal invitation from their treating medical professional from two Dutch hospitals (Cardiologist at the St. Antonius Hospital, Nieuwegein, The Netherlands; Nursing specialist at the Amsterdam UMC, VU University Medical Centre, Amsterdam, The Netherlands). The invitations were based on two criteria: the age of the patient (working age, between 18 and 67 years) and whether medical information had been requested by an occupational physician in the past six months. Sixteen invitees were willing to participate (St. Antonius *n* = 14, VUmc *n* = 2), of which nine met the inclusion criteria and were included in the study. Second, participants were recruited through the SSA. The SSA randomly invited a group of patients with CVD (*n* = 60) by sending them a letter to participate in the study. After two weeks, a reminder letter was sent. Ten positive responses were received, and eight of these respondents met the inclusion criteria and were included. In both recruitment strategies, the first or second author contacted interested patients by phone for further screening of the inclusion criteria. When the patient met all inclusion criteria, an online interview was scheduled. All participants provided written consent. Initially, the goal was to include fifteen participants to ensure reaching data saturation [35].

#### Participant characteristics

This study included 17 patients of which 14 males. The participants had a mean age of 53.8 (SD 11.2) years old and were in different stages after being diagnosed with CVD, experiencing various work participation problems. At the time of the interviews, the participants had a mean disease duration of 2.1 years (SD 1.4) since their CVD diagnosis. Prior to their diagnosis, the participants were either full-time (*n* = 9) or part-time (*n* = 8) working as a contracted employee (*n* = 15) or temporary worker (*n* = 2). At the time of the interview, some participants had fully returned to work (*n* = 6), had partly returned to work (*n* = 4), or had not (yet) returned to work (*n* = 7). For an overview of the demographic characteristics of the participants, see Table 2. For an overview of the proportions of participants over the PEJM and their position at the moment of the interview, see Supplementary material 1.

**Table 2:** Demographic characteristics of the participants (*n* = 17)

Variable	Mean (SD)	n	%
**Age, years**	53.8 (11.2)		
18–29		1	5.9
30–39		2	11.8
40–49		1	5.9
50–59		7	41.2
60–67		6	35.2
**Gender**			
Male		14	82.4
Female		3	17.6
**CVD diagnosis**			
Cardiac sarcoidosis		1	5.9
Endocarditis		1	5.9
Heart failure		2	11.8
Heart rhythm disorder		2	11.8
MINOCA		2	11.8
Pericarditis		2	11.8
Stroke (multiple)		7	41.2
**Time since diagnosis, years**^**a**^	2.1 (1.4)		
**Job sector**^**b**^			
Education and training		1	5.9
Engineering, production and construction		1	5.9
Healthcare and wellbeing		4	23.5
Security and public administration		3	17.6
Trade and services		3	17.6
Tourism, recreation and catering		1	5.9
Transport and logistics		4	23.5
**Type of work agreement**^**b**^			
Contracted employee		15	88.2
Temporary worker		2	11.8
**Number of hours working before CVD diagnosis**^**b**^			
Full-time, > 32 h		9	52.9
Part-time, ≤ 32 h		8	47.1
**Working status**^**a**^			
Fully returned to work		6	35.3
Partly returned to work		4	23.5
Not returned to work		7	41.2
**Duration sick-leave**^**c**^			
< 2 years sick leave		4	36.4
> 2 years sick leave (receiving benefit)		7	63.6

*CVD* Cardiovascular disease, *MINOCA* Myocardial infarction with non-obstructive coronary arteries, *SD* Standard deviation

^a^At the time of the interview

^b^At the time of diagnosis/start medical intervention

^c^When at partial or full-time sick leave at the time of the interview

### Data collection

#### Preparatory assignments

All participants were given preparatory assignments prior to the interview. The aim of these preparatory assignments was twofold: First, to stimulate, encourage and motivate participants to reflect on their experiences with work-focused healthcare in their own time and environment [27, 36]. Second, for the researchers to gain insight into the personal context of the participant prior to the interview, enabling them to delve further into specific topics during the interview [27, 36]. The preparatory assignments consisted of three tasks: 1) Listing all professionals they encountered during their (work-related) healthcare process; 2) Presenting changes in work participation after the onset of their CVD and identifying the healthcare professionals involved using a graphical timeline; 3) Listing all professionals who shared information or communicated about work (For the English translation of the full assignments, see Supplementary material 2). All participants received the preparatory assignments in hard copy at their home address and returned them via a pre-paid envelop before the interview. During the online interviews, PowerPoint slides were utilized to display the indicated timeline and list of professionals as supporting material.

#### The semi-structured interview

Semi-structured interviews (*n* = 17) of approximately one hour were conducted between February 2021 and July 2021, through a video call platform (Microsoft Teams). One interview was conducted through a telephone call, due to problems with the internet connection. An interview guide with listing topics and open-ended questions aiming to get insight into the patients’ journey and related experiences and needs was used as a memory aid for the interviewer during the interview (see Supplementary material 3). The interview guide and the use of the supporting materials were piloted twice with individuals recruited from the authors’ own network. These individuals experienced work participation problems, but their conditions were related to chronic diseases other than CVD. All interviews were conducted by the first (MH) and second author (NZ), alternating the role of the first and second interviewer. All interviews were performed in Dutch and were voice recorded with the permission of the participants. The voice recordings of the interviews were transcribed verbatim and de-identified for data analysis. The transcripts were sent back to each interviewee for member checking, allowing them to provide feedback concerning the completeness of the written material. Any additional follow-up questions of the researchers were shared with the interviewee following the interview and asked for a written response. These responses were added to the transcripts (*n* = 8). No repeat interviews were carried out.

### Data analysis

The graphical representation of the PEJM was created using the PEJM approach to analyse and map the patients’ experiences identified from the semi-structured interviews [37]. The development of the PEJM consisted of three steps: In step one, the first author (MH) analysed the interviews for segments containing the patient’s perspective on work-focused healthcare, checked by the second author (NZ). In step two, the third author (FO) aggregated these segments into the four different layers of information a PEJM exists of [38]: i) Phases and touchpoints, i.e. all stages that the patient goes through including all moments of contact with the healthcare system, ii) activities, i.e. what patients do to get their needs addressed, iii) positive and negative experiences that help or prevent patients from achieving their needs or goals, and iv) needs, i.e. a job to be done, a goal or need that the patient wants to have achieved. Subsequently, per phase a timespan indicating the elapsed time within a certain phase and all relevant stakeholders for that phase were defined [26]. The identified positive and negative experiences were aggregated within an emotion curve, substantiated with representative quotes showing a certain level of confirmability of the findings. The aggregated content of all components were iteratively developed, improving the PEJM, by discussion between the first three authors (MH, NZ, FO) to secure consistency. In step three, opportunities for improvement were derived from the aggregated data of the positive and negative experiences and the associated needs by the researchers (MH, NZ, FO) [39]. During subsequent sessions with almost all authors (MH, NZ, FO, JH, PW, CH, MM, SB), the researchers decided on the most important opportunities for improvement established based on a specific degree of significance.

The online platform Miro^1^, an online whiteboard for visual collaboration, was employed to aid the cluster and iteration process. The PEJM visualization was iteratively developed by the third author (FO) using Adobe Illustrator. The participants did not verify the findings. A thematic analysis of these interviews, presenting the findings on a more comprehensive analytical level, will be published elsewhere.

### Role of the researchers and ethical considerations

The first author (MH) had no prior experience with conducting qualitative research. However, the second author (NZ) is an experienced researcher in qualitative studies and took on the role of educating and supporting the first author. Additionally, the first author (MH) underwent a multi-day training to familiarize herself with qualitative studies and conducting interviews prior to the interviews of this study. Both MH and NZ are full-time researchers, without a background as (occupational) health experts, which helps minimize bias in the findings. The third author (FO) was involved as a research assistant and had experience in developing a PEJM. The other authors (JH, PW, CH, EC, MM, SB) are experienced researchers within the field of occupational health or human-centered design and helped shaping the study’s aim and relevance.

There were no established relationships between the interviewers and the participants prior to the study. Written consent was obtained after informing the patients about the objectives of the study. All participants received a small compensation for their time. The Medical Ethics Committee of the Amsterdam University Medical Centre granted ethical approval for the study. The committee declared that the study design did not require comprehensive ethical review, as the Medical Research Involving Human Subjects Act (‘Wet Medisch-wetenschappelijk Onderzoek met Mensen’) did not apply to this study (Reference number: W20_556 # 20.619).

## Results

**Fig. 1:**
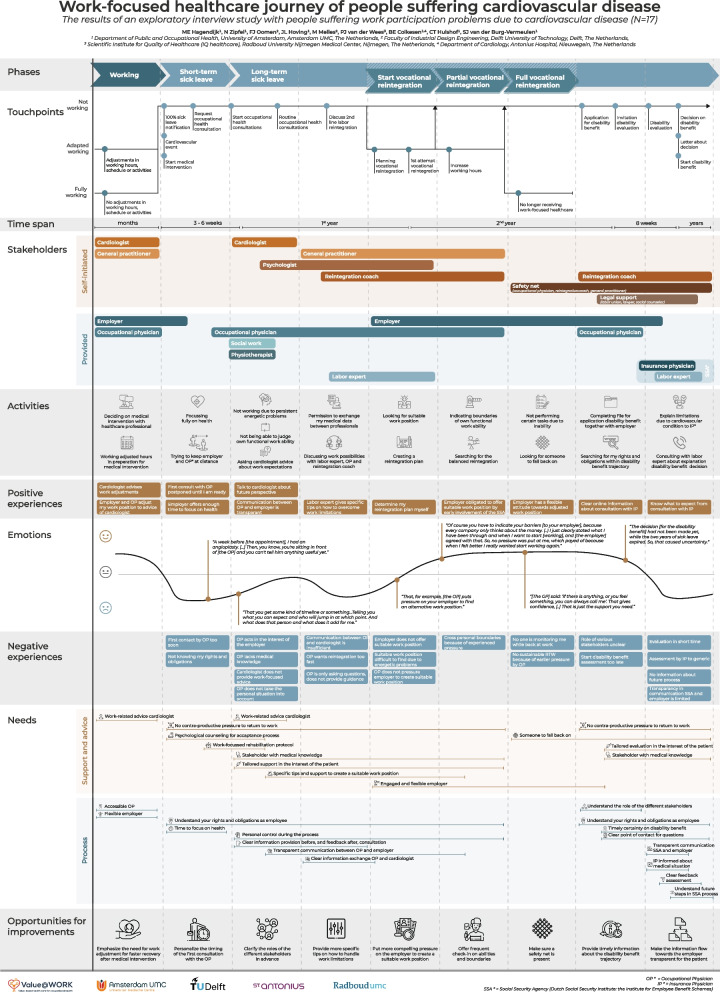
The work-focused healthcare journey of people living with cardiovascular disease. Figure 1 can also be opened in PdF via the Additional file 2 for better zooming options. Legends: Vertical axis show the multiple building blocks this Patient experience journey map exists of. Horizontal axis shows the data of the multiple building blocks over time. IP = Insurance physician, OP = Occupational physician, RTW = return to work, SSA = Social security agency

Figure 1 depicts the work-focused healthcare journey of people living with CVD that is aggregated based on all interview data. Based on the patients’ input six main phases are identified. The first phase, *i.e. working*, represents a period in which problems with health and functioning first occur. The next two phases, *i.e. short-term sick leave* and *long-term sick leave*, represent a period of full-time sick leave. The last three phases*, i.e. start vocational reintegration, partial vocational reintegration,* and *full vocational reintegration*, focus on the process of reintegration that takes place sometime within the two years after initial sick leave. This time frame is in concordance with the Dutch Gatekeeper Improvement Act, which provides guidelines for the employer and employee in order to get the sick-listed employee back to work as quickly as possible.

The six phases are described below, providing further explanations on related touchpoints, timespan, stakeholders, activities, experiences, emotions, and needs, as graphically represented in Fig. 1. The long-term sick leave phase is subdivided into four sub-phases (part 1–4) because of the long time span. The nine opportunities for improvement derived from the PEJM are highlighted at the end of the results section.

### Working

The working phase, pre-sick leave, contains two paths: adapted working and fully working. The adapted working path presents patients who knew they had CVD, and adapted their work, e.g. hours or activities, in preparation for their scheduled surgery *(see *Fig. 1*; touchpoints: adapted working, and activities).* These patients decided on, and waited for, their surgery in consultation with their general practitioner and cardiologist *(see *Fig. 1*; stakeholders and activities)*. During this phase patients experienced receiving work-focused advice from the cardiologist in preparation for the surgery to be positive *(see *Fig. 1*; positive experiences and needs)*.

*“I told the [cardiologist taking the intake for the surgery], I was working night shifts. And then he said: ‘You should stop [with working the night shifts], you just need to be in the best condition before surgery. (..) He explicitly gave me advice about [work].”* – pt 9

However, to put work-focused advice from the cardiologist into practice, patients indicated the need for a flexible employer, and an accessible occupational physician, for the realization of work adjustments *(see *Fig. 1*; stakeholders, positive experiences, and needs)*. The fully working path includes patients who reported no work adjustments because they had not yet experienced cardiovascular problems or were unaware of their underlying cardiovascular problem *(see *Fig. 1*; touchpoints: fully working)*.

### Short-term sick leave

All patients had a period of full-time sick leave at the onset of the cardiovascular event or the start of their medical intervention, e.g. surgery *(see *Fig. 1*; touchpoints)*. Following this onset of sick leave, the occupational physician contacted the patients for occupational health consultation *(see *Fig. 1*; touchpoints)*. A large share of the patients indicated that this first contact made by the occupational physician was too soon after the start of their medical intervention *(see *Fig. 1*; negative experiences)*, highlighting the need for some time to focus fully on their recovery and accept their medical condition and bodily impairments *(see *Fig. 1*; activities and needs)*.

*“The week before [the appointment with the occupational physician], I had an angioplasty. (..) Then, you know, you’re sitting in front of [the occupational physician] and you can’t tell him anything useful yet.”* – pt 2

Therefore, in this phase, patients indicated that they tried to avoid any formal contact with the employer and occupational physician *(see *Fig. 1*; activities).* Employers offering enough time to focus on recovery and postponing the consultation with the occupational physician contributed positively to this process of acceptance *(see *Fig. 1*; positive experiences)*. Patients mentioned psychological counselling and a work-focused rehabilitation protocol to be important *(see *Fig. 1*; needs)*. However, psychological consultation often was not provided, resulting in self-initiated psychological consultation later in time *(see *Fig. 1*; stakeholders: self-initiated)*. In addition, patients expressed the need to understand their rights and obligations during sick leave *(see *Fig. 1*; needs and negative experiences)*.

*“At the moment you come home [after hospitalization], one of the most annoying things is that you are not aware of your rights [as an employee]. You do not know what is coming next and what you can do to stand up for yourself.”* – pt 6

### Long-term sick leave

#### Part 1—first few months

After approximately six weeks of sick leave, the first consultation with the occupational physician took place *(see *Fig. 1*; timespan and touchpoints)*. The occupational physician supported patients in finding the optimal work position, matching their energetic limitations, and understanding the consequences for their functional work ability *(see *Fig. 1*; activities)*. However, patients expressed the need for more medical knowledge, more tailored support in the interest of the patient, and no counterproductive pressure for vocational reintegration during consultation with the occupational physician *(see *Fig. 1*; negative experiences and needs)*. Besides discussing future work ability with the occupational physician, patients indicated to highly value work-focused advice from their cardiologist *(see *Fig. 1*; positive experiences and needs)*. Herefore, some patients asked for work-related advice from the cardiologist *(see *Fig. 1*; activities and negative experiences)*.

*“The cardiologist knows exactly what my diagnosis means. I like that, if I ask [my cardiologist] about what I can do [regarding work activities], you get an answer that you can rely on. You can take [the cardiologist's] at his word.”* – pt 1

According to the process of consultations, patients mentioned to value a clear information provision before, and feedback after consultation with professionals involved in their work-focused healthcare.  In addition, the patients highlighted the need for transparency in the communication from their healthcare professionals towards the employer *(see *Fig. 1*; positive experiences and needs)*.

*"I would prefer to receive a summary [of the consultation with the occupational physician], including what we are going to do in the future, what [the occupational physician] is expecting, and his vision. I would really like to know that." –* pt 9

#### Part 2—towards the end of the 1^*st*^* year*

The long-term sick leave phase continued with discussing work possibilities during routine occupational health consultations and 2^nd^ line labor expert consultations, exploring alternative work positions outside the current job sector *(see *Fig. 1*; touchpoints and activities)*. Patients experienced counterproductive pressure for vocational reintegration when the occupational physician put pressure on vocational reintegration too fast *(see *Fig. 1*; negative experiences and needs)*. Besides, patients highlighted the need for specific guidance on how to overcome any work limitations *(see *Fig. 1*; positive experiences and needs)*, since only answering the occupational physicians’ questions regarding work limitations and expectations were experienced negative *(see *Fig. 1*; negative experience)*.

*"You do not get any guidance, [the occupational physician] only asks you questions."* – pt 2.

In this phase, patients also expressed the need for clear information exchange between the occupational physician and cardiologist, for which the patients gave consent *(see *Fig. 1*; activities)*. This information exchange was often experienced as insufficient due to long waiting times or incorrect information *(see *Fig. 1*; negative experiences and needs)*.

*“The occupational physician did ask the cardiologist [via a letter] about my diagnosis and what restrictions the cardiologist did impose on me. Then the cardiologist answered: ‘I did not impose any restrictions on the patient’. Which is true, the cardiologist did not do that, but my body did. But the occupational physician then was convinced I could work again.”* – pt 10

#### Part 3—towards the end of the 2^*nd*^* year*

When (full) vocational reintegration was not possible or successful, patients applied for a disability benefit at the SSA in collaboration with their employer towards the end of the second year of (partial) sick leave *(see *Fig. 1*; touchpoints and activities)*. At this point, the need for a better understanding of the role of the stakeholders was mentioned *(see *Fig. 1*; needs)*, since a lack of understanding is a bottleneck for the patients to properly prepare for the SSA trajectory *(see *Fig. 1*; negative experiences)*.

*“When applying for the disability benefit, at that moment I realized that I actually had no idea how the system works. (..) I felt like it would be useful at this point if I had a better understanding of which professional played with role in my process.”* – pt 14

Following the application, patients were invited for disability evaluation by the insurance physician and the labor expert from the SSA *(see *Fig. 1*; touchpoints)*. Patients identified being satisfied with the provided information about the upcoming consultations with the insurance physician *(see *Fig. 1*; positive experiences)*. In case of any remaining questions, the need for a clear point of contact was highlighted *(see *Fig. 1*; needs)*.

In addition, patients found it of great importance to have timely certainty on the outcome of the disability benefit assessment *(see *Fig. 1*; needs)*. Planning the disability evaluation too late could cause a lot of uncertainty regarding future income *(see *Fig. 1*; negative experiences and emotions)*.

*“The decision [for the disability benefit] had not been made yet, while the two years of sick leave expired. So, that caused uncertainty.”* – pt 15

Subsequently, patients searched continuously for their rights and obligations in preparation for, and during the disability evaluation process *(see *Fig. 1*; activities)*. Therefore, patients regularly engaged stakeholders, such as a labor union, a lawyer or social counsellor, to provide legal support *(see *Fig. 1*; stakeholders)*.

#### Part 4—after the 2^*nd*^* year*

During the disability evaluation by the insurance physician working for the SSA, patients explained their functional limitations in daily life and participation due to their cardiovascular condition *(see *Fig. 1*; touchpoints and activities)*. However, patients often felt insecure during consultation with the insurance physician, due to limited time, standardized protocols, and the feeling that the insurance physician was not sufficiently informed about their medical situation prior to the evaluation *(see *Fig. 1*; negative experiences and needs)*.

*“The insurance physician decides the percentage of work disability based on standard protocols. If you are a heart patient, they take a look in the protocol and it describes a percentage. It is the same for all heart patients. Which makes me wonder if they really assess the personal situation.”* – pt 6

Subsequently, patients were informed about the decision regarding the disability benefit during consultation with the labor expert, and received a letter about the decision afterwards *(see *Fig. 1*; touchpoints and activities)*. Patients highlighted lacking transparency in communication between the SSA and the employer regarding this decision *(see *Fig. 1*; negative experiences and needs).*

*“But I do not know if my employer (..) received some kind of report [from the SSA about the decision of the disability benefit]. I have no idea, but I hope that was the case.”* – pt 14

After granting the disability benefit *(see *Fig. 1*; touchpoints)*, patients experienced a lack of information provided regarding the future disability benefit trajectory *(see *Fig. 1*; negative experiences and needs)*. Besides, the need for someone to fall back on remained *(see *Fig. 1*; needs)*.

### Start vocational reintegration

When planning vocational reintegration, patients looked for a suitable work position matching their functional limitations supported by the labor expert, occupational physician, and eventually the reintegration coach *(see *Fig. 1*; touchpoints, stakeholders, and activities)*. With the support of these stakeholders, a reintegration plan was created *(see *Fig. 1*; activitie*s), in which patients expressed their own decision-making to be important *(see *Fig. 1*; positive experiences)*. Subsequently, patients consulted the reintegration coach and general practitioner to identify the boundaries in functional work ability and balance between working and private life *(see *Fig. 1*; activities and stakeholders)*. However, finding a suitable work position could be difficult and employers did not always show flexibility and engagement by offering adjusted work positions *(see *Fig. 1*; negative experiences and needs)*. When the employer lacked this flexibility and engagement, patients expressed the need for work-focused healthcare professionals to put pressure on the employer to stimulate to create a suitable work position *(see *Fig. 1*; negative experiences and needs)*.

*"I expected that the SSA would chase the employer [when the employer does not fulfill its obligations]. (..) But that did not happen."* – pt 13

Creating a suitable work position was followed by the first attempt at vocational reintegration *(see *Fig. 1*; touchpoints)*.

### Partial vocational reintegration

When the first attempt for vocational reintegration was successful, working hours were increased *(see *Fig. 1*; touchpoints)*. Here, patients highlighted searching for a balanced vocational reintegration *(see *Fig. 1*; activities)*, in which again the patients appreciated efforts by the employe, the occupational physician or the SSA to create a sustainable work position *(see *Fig. 1*; positive experiences and needs)*. Also, patients indicated their functional boundaries to the employer and occupational physician, to protect themselves from any counterproductive pressure and prevent relapse to full-time sick leave *(see *Fig. 1*; activities and negative experiences)*.

*“Of course you have to indicate your barriers [to your employer], because every company only thinks about the money. (..) I just clearly stated what I have been through and what I’m feeling and when I want to start [working]. (..), and they [the employer] agreed with that. So, no pressure was put at me, which payed of because when I felt better I really wanted start working again.”* – pt 18

### Full vocational reintegration

When patients succeeded to build up working hours, the next and final step was full vocational reintegration *(see *Fig. 1*; touchpoints)*. During this phase, patients could still not perform certain tasks due to chronic bodily impairments *(see *Fig. 1*; activities).* Therefore, patients mentioned an engaged and flexible attitude from the employer to be valuable *(see *Fig. 1*; positive experiences and needs)*.

*“[The employer] took the moments of stress during work away, by limiting my number of customers. (..) Furthermore, I do not lift heavy boxes by using special equipment for that. Those were the adjustments made [by my employer] to help me get fully back to work.”* – pt 5

Patients felt insecure because no one was monitoring them while back at work *(see *Fig. 1*; negative experiences)*, and expressed the need for someone to fall back on *(see *Fig. 1*; needs and activities).* Patients welcomed the offer from various stakeholders, e.g. the occupational physician, reintegration coach, or medical specialist, to contact them whenever needed *(see *Fig. 1*; stakeholders and emotions)*.

*“[The GP] said: ‘if there is anything, or you feel something, you can always call me’. That gives confidence, (..) That is just the support you need.”* – pt 11

### Opportunities for improvement to better meet the patient’s needs

Opportunities to improve work-focused healthcare from patients’ perspectives were identified throughout the various work-focused healthcare phases, based on the experiences and needs of the patients. Below, nine opportunities for improvement and their impact are presented in the order in which they appear in the PEJM (*see *Fig. 1*, final row; opportunities for improvement*).

#### Emphasize the need for work adjustment for faster recovery after medical intervention (phase: working):

Urging the need for, and supporting patients in, adjusting their work (work tasks and/or work environment) before medical intervention by involved professionals may contribute to a faster recovery and thus faster vocational reintegration.

#### Personalize the timing of the first consultation with the occupational physician (phase: short-term sick leave):

The large variety in the personal situation, and therefore, the timing of readiness to talk with occupational health professionals requests for adjusting the timing of the first consultation to the personal situation which may prevent the feeling of counterproductive pressure and rush.

#### Clarify the roles of the different stakeholders in advance (phase: long-term sick leave, part 1):

 Improving information provision regarding the role of stakeholders towards the patients may facilitate less uncertainty and more autonomy at a later moment in time during the work-focused healthcare process.

#### Provide advice on how to handle work limitations (phase: long-term sick leave, part 2):

 Offering the patients more specific tips on how to deal with their functional limitations during work, including tips regarding adjustments in work demands, working hours or workplace, may give the patients better ability and self-efficacy for vocational reintegration.

#### Put more compelling pressure on the employer to create a suitable work position (phase: start vocational reintegration):

Putting more pressure on the employers to offer opportunities for adjustments in work position, may facilitate a faster patient’s vocational reintegration.

#### Offer frequent check-in on abilities and boundaries (phase: partial vocational reintegration):

 Offering more frequent check-ins with professionals to discuss work throughout the patient’s journey, supporting the search for a balanced reintegration and setting personal boundaries, may support the patient’s vocational reintegration.

#### Make sure a safety net is present (phase: full vocational reintegration):

 Offering the patients the opportunity for continuity in support after full vocational reintegration or during the disability benefit, may prevent relapse and even potentially allow the patients to build up working hours further in some cases.

#### Provide timely information about the disability benefit trajectory (phase: long-term sick leave, part 3):

 Clear and timely information on all process steps within the SSA trajectory, including the timeline of the disability benefit and reassessments, role of stakeholders and possibilities for reintegration support, may give the patients better knowledge of what to expect which can result in higher satisfaction levels.

#### Make the information flow towards the employer transparent for the patient (phase: long-term sick leave, part 4):

A more transparent information flow towards the employer, may give the patient more insight into, and a better understanding of, the employer’s actions.

## Discussion

In this qualitative study, we aimed to gain insight into the work-focused healthcare journey from the perspective of patients with work participation problems due to a CVD, to understand their experiences and needs, and to derive opportunities from these experiences and needs for improving work-focused healthcare service at a system level. The work-focused healthcare journey as perceived by these patients was explored using the PEJM approach, which enabled us to identify multiple phases within the work-focused healthcare system, along with related touchpoints, timespan, stakeholders, activities, positive and negative experiences, emotions, and needs. Six main phases were identified in the patients’ work-focused healthcare journey: working, short-term sick leave, long-term sick leave, start vocational reintegration, partial vocational reintegration and full vocational reintegration. While we found various inconsistencies between the identified experiences and needs in the data, we derived nine opportunities for improvement being most significant for each (part of a) phase, in order to convey a clear message for practice. These opportunities included, among others, adjusting consultation timing, improve information provision and exchange over time, provide more personalized advice on handling work limitations, and put more compelling pressure on the employer to create suitable work positions for their employees.

A broad and holistic understanding of the work-focused healthcare system over time and place from the patients’ perspective is the starting point to identify bottlenecks and opportunities for patient-centered improvements in the healthcare process [27]. While previous literature has discussed similar experiences with work-focused healthcare for both patients living with CVD [13, 14, 19, 40–42] and other chronic conditions [43–45], to our knowledge, a graphical time-bound representation of the patients’ full work-focused healthcare journey, including their experiences and needs over time and place, was not presented before. Consequently, earlier literature did not systematically identify opportunities for improvement to better meet the patients’ needs within work-focused healthcare. However, this method of deriving opportunities for improvement from PEJM data has been previously employed in studies enhancing patient experiences in healthcare [46].

Looking into the individual opportunities for improvement identified in this study, the opportunity describing the provision of more specific person-oriented advice on how to handle work limitations is supported by earlier literature, since addressing their individual needs is appreciated by patients [47], and may result in better patient-satisfaction and quality of care [48]. The opportunities for improvement related to information provision to patients, such as timely clarifying the roles of the different stakeholders and providing timely information about the disability benefit trajectory, are grounded in patients’ expressed needs for predictability, control, and security during their sick leave, as reported in previous literature [49]. Similar needs for clear information on the roles of healthcare professionals and disability benefits were also observed in studies focusing on other patient populations, as patients with acquired brain injury and cancer [50–52]. To ensure comprehensive information provision during sick-leave, additional practices can be employed, such as involving the employer in providing necessary information [53], or designating a coordinator in the RTW process to guide patients [54].

Moreover, the opportunity to enhance work-focused healthcare by placing greater emphasis on the importance of appropriate workplace adjustments, is supported by earlier literature describing the need for pre-surgery education and RTW planning for patients living with CVD [55]. Additionally, medical specialists’ advice and assessment regarding work-related matters were found to be trusted by patients [56]. However, earlier literature indicated that medical specialists often face constraints in discussing work-related issues with patients or occupational physicians due to limited capacity and time [57]. Furthermore, patients’ preferences for personalized timing of the first consultation with the occupational health professional, frequent check-ins, and the presence of a safety net were also earlier identified in studies focussing on other patient populations [58–60]. In addition, in this study, patients reported limited flexibility of the timing of the first and follow-up consultations, being a hindrance to meet individual needs effectively. The lack of availability and flexibility in work-focused healthcare could be attributed to the existing shortage of occupational healthcare professionals [61, 62] and the minimal standard support mandated by the Dutch legislations [31]. Furthermore, existing literature highlighted a lack of unity among the multiple stakeholders within work-focused healthcare. For example, earlier literature reported an employers’ preference for financial advantageous acknowledged by occupational physicians and a lack of visibility of occupational physicians indicated by employers [61, 63]. To improve this unity among stakeholders, it is suggested to stimulate involvement and transparency between the multiple stakeholders within work-focused healthcare [64, 65].

### Methodological considerations

Although the application of the PEJM approach in work-focused healthcare is quite novel, it is a well-established system approach to visualize the dynamics of a sociotechnical healthcare system over time [66, 67]. The use of multiple layers identified and visually represented per phase provides a comprehensive overview of patients’ perspectives in their work-focused healthcare journey. To create an aggregated PEJM, representing only the most relevant findings from the data, a specific degree of significance was assigned based on the interpretations and experiences of the authors [38]. The study reached data saturation, as no new theoretical insights emerged during the analysis of the last two interviews.

Given the retrospective character of the interviews and the average of more than two years post-diagnosis, there might be a certain level of recall bias. However, efforts were made to restrain the influence of recall bias on the findings by the use of the preparatory assignments within the study design [36]. Additional prospective observations are suggested to develop a more complete understanding of patients work-focused healthcare journey [66]. It is important to acknowledge that our sample of participants may skewed towards patients with a specific interest in the topic or those with overly positive or negative experiences with work-focused healthcare. This bias could potentially result in a lack of negative experiences and subsequently missed opportunities for improvement if the sample is overly positive, or a lack of positive experiences if the sample is overly negative with derived opportunities for improvement limited to a small group. However, we did not observe a disproportionate distribution between positive and negative experiences. Nonetheless, there remains some uncertainty regarding the potential influence of selection bias on our results since information on non-responder characteristics was unavailable due to privacy regulations. Besides, excluding non-Dutch speakers, the use of preparatory assignments and online interviewing might have contributed to a selection bias in favour of native Dutch speakers and digitally educated participants. As a result, the experiences and needs of non-(native) Dutch speakers and lower digital literacy may be underrepresented. In total, 17 interviews were conducted, including participants with a large range of disease duration and current working status, ensuring data saturation and transferability. However, workers with temporary contracts were underrepresented in our sample [68], which may limit the generalizability of the findings to this group of employees. The skewed distribution between contracted and temporary employees might be explained by the recruitment strategy, which involved sampling patients for whom medical information was requested by an occupational physician, leaving out temporary workers who might have applied to The Sickness Benefits Act with work-focused healthcare provided by an insurance physician after losing their employment contract while on sick leave. Moreover, few women were included in this study, which may partly be explained by the smaller amount of women diagnosed with CVD [69]. This may impact the generalizability of the findings, given the gender-specific differences in prioritizing work and social roles [70]. The member checking, pilot testing and validity checks within the research team ensured the credibility and trustworthiness of the data [71].

### Implications for future research

In this study we conducted a qualitative study, limiting the generalizability to a broader population of employees experiencing work participation problems due to CVD. To establish greater generalizability, additional quantitative research could be conducted to validate the identified experiences, needs and effects of interventions based on the opportunities for improvement across a wider range of individuals. It is important to note that our study only included employees, not taking into account the position of the self-employed workers in the work-focused healthcare context. The generalizability of our PEJM to self-employed workers might be limited due to differences in access to occupational healthcare and work disability insurance. Therefore, further research is needed to explore the experiences and needs of self-employed workers for work-focused healthcare to identify potential opportunities for improvement. Although this study successfully identified and visualized the work-focused healthcare journey for patients living with CVD, it may be interesting to study the generalizability of the findings to a broader range of chronic diseases. Previous literature on other patient populations suggests that similar experiences and needs might be prevalent [72–75]. It is expected that implementation of interventions addressing the identified opportunities for improvement may face various barriers, such as (privacy) laws and financing issues for personalizing consultation time or enhancing information exchange transparency. Although it is anticipated that incorporating these interventions would enhance the experiences of patients within work-focused healthcare [76], this is not yet confirmed. Further research is required to investigate the possibilities and challenges of implementing interventions targeting the suggested opportunities for improvement and their impact on the experiences of patients, as well as other stakeholders, including a.o. occupational and insurance physicians. Additionally, it would be valuable to explore the perceived barriers and facilitators experienced by professionals while striving to provide patient-centered work-focused healthcare. Understanding these factors could help identify further opportunities to improve patient-centered work focused healthcare.

### Implications for practice

Although this research was conducted in the specific jurisdiction of the Netherlands, the findings related to patients’ activities, experiences, needs and opportunities for improvement targeting the general work-focused healthcare system are likely being transferable to healthcare contexts outside the Netherlands. For instance, the opportunities for improvement aiming at better patient-centered work-focused healthcare systems, such as personalizing the timing of the first consultation with the occupational healthcare professional or providing a safety net after full RTW, may also hold relevance for healthcare systems in other countries [41, 42]. Moreover, the aspects of time and place in relation to the patient’s perspective, characterizing the PEJM approach, are in line with the current focus in healthcare towards integrated patient-centered delivery of care at the right time and place [77, 78]. Insight into the patients’ experiences over time and place empowers professionals within the work-focused healthcare journey and guideline makers to continuously improve the patient-centeredness within the system. Some of the suggested opportunities for improvement can be immediately implemented by professionals in their provided services. For instance, clinical care professionals can emphasize the need for work adjustment prior to medical intervention, while occupational health professionals can provide more personalized advice on handling work limitations. Additionally, other opportunities for improvement, such as adjusting consultation timing and offering a safety net, may serve as an impetus for guideline makers to design work-focused healthcare systems more patient-oriented. Understanding the patients’ work-focused healthcare journey and the corresponding opportunities for improvement promotes creative thinking to reform work-focused healthcare and facilitates meaningful dialogue within and between the multiple stakeholder groups searching for solutions [79]. Moreover, the PEJM approach facilitates professionals with insights into the involvement and activities of other stakeholders, which promotes better collaboration among multiple stakeholders [80]. Work-focused healthcare providers can use the insights from this PEJM during shared decision-making with patients, enabling discussions that revolve around more patient-centered outcomes [76].

## Conclusion

This paper makes a significant contribution to enhancing a more patient-centered work-focused healthcare system for patients employed in paid jobs when living with CVD. It achieves this by providing a comprehensive understanding of the patients’ perspectives throughout their work-focused healthcare journey and highlighting opportunities for improvement over time and place. For instance, the identified opportunities for improvements emphasize the need for work adjustment for faster recovery after medical intervention. Additionally, there is a call for improving information provision and exchange over time. Besides, providing specific person-oriented advice on how to handle work limitations is also deemed crucial. Moreover, putting more compelling pressure on the employer to create a suitable work position is identified as a vital area of improvement.

### Supplementary Information

**Additional file 1: Supplementary material 1.** Proportion and position of the participants. **Supplementary material 2.** The sensitizing booklet. **Supplementary material 3.** Interview guide.

**Additional file 2.** The work-focused healthcare journey of people living with cardiovascular disease. Legends: Vertical axis show the multiple building blocks this Patient experience journey map exists of. Horizontal axis shows the data of the multiple building blocks over time. IP = Insurance physician, OP = Occupational physician, RTW = return to work, SSA = Social security agency

## Data Availability

The datasets used and/or analysed during the current study are available from the corresponding author on reasonable request.

## Abbreviations

CVD: Cardiovascular disease
PEJM: Patient experience journey map
RTW: Return to work
COREQ: Consolidated criteria for reporting qualitative research
SSA: Social security agency

## Footnotes

1 https://miro.com

## References

[1] Luepker RV (2011). Cardiovascular disease: rise, fall, and future prospects. Annu Rev Public Health.

[2] WHO. Cardiovascular diseases (CVDs). Available from: https://www.who.int/news-room/fact-sheets/detail/cardiovascular-diseases-(cvds).

[3] Hart- en vaatziekten | Leeftijd en geslacht [Cardiovascular disease | Age and gender] 2021 Available from: https://www.vzinfo.nl/onderwerpen/hart-en-vaatziekten/leeftijd-en-geslacht.

[4] Pilipiec P, Groot W, Pavlova M (2021). The effect of an increase of the retirement age on the health, well-being, and labor force participation of older workers: a systematic literature review. Journal of Population Ageing.

[5] Chichkanov VP, Chistova EV, Tyrsin AN, et al. Consequences of raising the retirement age for the labor market in the regions of russia. 2019.

[6] Rabate S, Rochut J (2020). Employment and substitution effects of raising the statutory retirement age in France. J Pension Econ Finance.

[7] Dekkers-Sánchez PM, Wind H, Sluiter JK (2010). A qualitative study of perpetuating factors for long-term sick leave and promoting factors for return to work: chronic work disabled patients in their own words. J Rehabil Med.

[8] Tella NC, Arnaiz CS, Gatius JR (2017). Assessment of the length of sick leave in patients with ischemic heart disease. BMC Cardiovasc Disord.

[9] de Rijk A. Coronary heart disease and return to work. Springer; 2020.

[10] Salzwedel A, Koran I, Langheim E (2020). Patient-reported outcomes predict return to work and health-related quality of life six months after cardiac rehabilitation: Results from a German multi-centre registry (OutCaRe). PLoS ONE.

[11] Gragnano A, Negrini A, Miglioretti M (2018). Common psychosocial factors predicting return to work after common mental disorders, cardiovascular diseases, and cancers: a review of reviews supporting a cross-disease approach. J Occup Rehabil.

[12] Huber M, Knottnerus JA, Green L, et al. How should we define health? Bmj. 2011;343. 10.1136/bmj.d4163 21791490

[13] Blokzijl F, Onrust M, Dieperink W (2021). Barriers that obstruct return to work after coronary bypass surgery: a qualitative study. J Occup Rehabil.

[14] Gard G, Pessah-Rasmussen H, Brogårdh C (2019). Need for structured healthcare organization and support for return to work after stroke in Sweden: Experiences of stroke survivors. J Rehabil Med.

[15] Bartys S, Stochkendahl MJ. Section 10, Chapter 12: Work-focused Healthcare for Low Back Pain.

[16] van Dijk J. De multidisciplinaire richtlijn Hartrevalidatie 2011. TBV–Tijdschrift voor Bedrijfs-en Verzekeringsgeneeskunde. 2011;19:410–415.

[17] Sadeghi M, Rahiminam H, Amerizadeh A (2022). Prevalence of return to work in cardiovascular patients after cardiac rehabilitation: a systematic review and meta-analysis. Curr Probl Cardiol.

[18] DE BEDRIJFSARTS RV. NVAB-richtlijn ‘Handelen van de bedrijfsarts bij werknemers met ischemische hartziekten’.

[19] Hartke RJ, Trierweiler R, Bode R (2011). Critical factors related to return to work after stroke: a qualitative study. Top Stroke Rehabil.

[20] Schweigert M, McNeil D, Doupe L (2004). Treating physicians’ perceptions of barriers to return to work of their patients in Southern Ontario. Occup Med.

[21] Bosma A, Boot C, Schaafsma F (2020). Facilitators, barriers and support needs for staying at work with a chronic condition: a focus group study. BMC Public Health.

[22] Dorland H, Abma F, Roelen C (2016). Factors influencing work functioning after cancer diagnosis: a focus group study with cancer survivors and occupational health professionals. Support Care Cancer.

[23] Vooijs M, Leensen MC, Hoving JL (2017). Perspectives of people with a chronic disease on participating in work: a focus group study. J Occup Rehabil.

[24] Ose SO, Kaspersen SL, Leinonen T (2022). Follow-up regimes for sick-listed employees: A comparison of nine north-western European countries. Health Policy.

[25] Melles M, Albayrak A, Goossens R (2021). Innovating health care: key characteristics of human-centered design. Int J Qual Health Care.

[26] Carayon P, Albayrak A, Goossens R (2021). Macroergonomics of patient work: engaging patients in improving sociotechnical context of their work.

[27] Simonse L, Albayrak A, Starre S (2019). Patient journey method for integrated service design. Design for Health.

[28] MacEachen E. The science and politics of work disability prevention. Routledge; 2018.

[29] KNMG. KNMG-visiondocument care that works: To a better work-oriented medical care for (potential) workers [KNMG- visiedocument zorg die werkt: naar een betere arbeidsger-ichte medische zorg voor (potentieel) werkenden]. Utrecht: KNMG; 2017.

[30] Business.gov.nl. Reintegration obligations. Available from: https://business.gov.nl/regulation/reintegration-obligations/.

[31] Business.gov.nl. Health and safety at work. Available from: https://business.gov.nl/regulation/health-safety-work/.

[32] Verbeek J, Spelten E, Kammeijer M (2003). Return to work of cancer survivors: a prospective cohort study into the quality of rehabilitation by occupational physicians. Occup Environ Med.

[33] Steenbeek R, Schellart AJ, Mulders H (2011). The development of instruments to measure the work disability assessment behaviour of insurance physicians. BMC Public Health.

[34] Tong A, Sainsbury P, Craig J (2007). Consolidated criteria for reporting qualitative research (COREQ): a 32-item checklist for interviews and focus groups. Int J Qual Health Care.

[35] Patten ML, Galvan MC (2019). Sampling in Qualitative Research.

[36] Visser FS, Stappers PJ, Van der Lugt R (2005). Contextmapping: experiences from practice. CoDesign.

[37] Trebble TM, Hansi N, Hydes T, et al. Process mapping the patient journey: an introduction. Bmj. 2010;341. 10.1136/bmj.c4078 20709715

[38] Van Boeijen A, Daalhuizen J, Zijlstra J. Delft Design Guide: Perspectives, models, approaches, methods. BIS Publishers; 2020.

[39] Moon H, Han SH, Chun J (2016). A design process for a customer journey map: a case study on mobile services. Hum Factors Ergon Manuf Serv Ind.

[40] Pourhabib A, Sabzi Z, Yazdi K (2022). Facilitators and barriers to return to work in patients after heart surgery. J Educ Health Promot.

[41] Lock S, Jordan* L, Bryan K, et al. Work after stroke: focusing on barriers and enablers. Disability & society. 2005;20(1):33–47.

[42] Hellman T, Bergström A, Eriksson G (2016). Return to work after stroke: Important aspects shared and contrasted by five stakeholder groups. Work.

[43] Kluit L, de Wind A, Oosting IJ, et al. Current practices, needs, and expectations of discussing work with a medical specialist from a patient’s perspective: a qualitative study. Disability and Rehabilitation. 2022:1–14. 10.1080/09638288.2022.2157500 36564948

[44] Olischläger DL, den Boer LXY, de Heus E, et al. Rare cancer and return to work: experiences and needs of patients and (health care) professionals. Disability and Rehabilitation. 2022:1–12. 10.1080/09638288.2022.2099589 35850601

[45] Joosen MC, Lugtenberg M, Arends I, et al. Barriers and facilitators for return to work from the perspective of workers with common mental disorders with short, medium and long-term sickness absence: a longitudinal qualitative study. Journal of Occupational Rehabilitation. 2021:1–12. 10.1007/s10926-021-10004-9 PMC9232415 34580811

[46] Philpot LM, Khokhar BA, DeZutter MA (2019). Creation of a patient-centered journey map to improve the patient experience: a mixed methods approach. Mayo Clin Proc Innov Qual Outcomes.

[47] Youssef A, Wiljer D, Mylopoulos M (2020). “Caring About Me": a pilot framework to understand patient-centered care experience in integrated care-a qualitative study. BMJ Open.

[48] Wolf DM, Lehman L, Quinlin R (2008). Effect of patient-centered care on patient satisfaction and quality of care. J Nurs Care Qual.

[49] Sturesson M, Edlund C, Falkdal AH (2014). Healthcare encounters and return to work: a qualitative study on sick-listed patients’ experiences. Prim health Care Res & Dev.

[50] Watter K, Kennedy A, McLennan V (2022). Consumer perspectives of vocational rehabilitation and return to work following acquired brain injury. Brain Impairment.

[51] Berger I, Beck L, Jones J (2020). Exploring the needs of cancer survivors when returning to or staying in the workforce. J Occup Rehabil.

[52] Yarker J, Munir F, Bains M (2010). The role of communication and support in return to work following cancer-related absence. Psychooncology.

[53] de Rijk A, Amir Z, Cohen M, et al. The challenge of return to work in workers with cancer: employer priorities despite variation in social policies related to work and health. J. 2020;14:188–199. 10.1007/s11764-019-00829-y PMC7182537 31758518

[54] Öst Nilsson A, Eriksson G, Johansson U (2017). Experiences of the return to work process after stroke while participating in a person-centred rehabilitation programme. Scand J Occup Ther.

[55] O'Brien L, McKeough C, Abbasi R (2013). Pre-surgery education for elective cardiac surgery patients: A survey from the patient's perspective. Aust Occup Ther J.

[56] Lysaght RM, Larmour-Trode S (2008). An exploration of social support as a factor in the return-to-work process. Work.

[57] Scharf J, Angerer P, Müting G (2020). Return to work after common mental disorders: a qualitative study exploring the expectations of the involved stakeholders. Int J Environ Res Public Health.

[58] Hubertsson J, Petersson IF, Arvidsson B (2011). Sickness absence in musculoskeletal disorders-patients' experiences of interactions with the social insurance agency and health care A qualitative study. BMC Public Health.

[59] Nouri F, Coole C, Baker P (2020). Return to work advice after total hip and knee replacement. Occup Med.

[60] Fassier J-B, Lamort-Bouché M, Broc G (2018). Developing a return to work intervention for breast cancer survivors with the intervention mapping protocol: challenges and opportunities of the needs assessment. Front Public Health.

[61] Harrison J, Dawson L (2016). Occupational health: Meeting the challenges of the next 20 years. Saf Health Work.

[62] Rijksoverheid. dit najaar maatregelen tegen wachtlijsten bij UWV [English: This fall measures against waiting lists of the SSA] Available from: https://www.rijksoverheid.nl/actueel/nieuws/2022/08/26/dit-najaar-maatregelen-tegen-wachtlijsten-bij-uwv.

[63] Bosma A, Boot C, Snippen N (2021). Supporting employees with chronic conditions to stay at work: perspectives of occupational health professionals and organizational representatives. BMC Public Health.

[64] Bosma A, Boot C, Schaafsma F (2020). Development of an intervention to create a supportive work environment for employees with chronic conditions: an intervention mapping approach. J Occup Rehabil.

[65] Greidanus M, de Rijk A, de Boer A (2021). A randomised feasibility trial of an employer-based intervention for enhancing successful return to work of cancer survivors (MiLES intervention). BMC Public Health.

[66] de Ridder EF, Dekkers T, Porsius JT (2018). The perioperative patient experience of hand and wrist surgical patients: an exploratory study using patient journey mapping. Patient Exp J.

[67] Manchaiah VK, Stephens D, Meredith R (2011). The patient journey of adults with hearing impairment: the patients’ views. Clin Otolaryngol.

[68] Statistiek_CBv. Aantal flexibele contracten met zekerheid in eerste kwartaal toegenomen [Number of flexible contracts increased in the first quarter of this year] Available from: https://www.cbs.nl/nl-nl/nieuws/2022/20/aantal-flexibele-contracten-met-zekerheid-in-eerste-kwartaal-toegenomen. 2022.

[69] Statistiek_CBv. Ziekteverzuim oudere werknemer zonder aandoening vrijwel even hoog als van jongere 2014 Available from: https://www.cbs.nl/nl-nl/nieuws/2014/27/ziekteverzuim-oudere-werknemer-zonder-aandoening-vrijwel-even-hoog-als-van-jongere.

[70] Emslie C (2005). Women, men and coronary heart disease: a review of the qualitative literature. J Adv Nurs.

[71] Cope DG, editor Methods and meanings: credibility and trustworthiness of qualitative research. Oncology nursing forum; 2014. 10.1188/14.ONF.89-91 24368242

[72] Aamland A, Werner EL, Malterud K (2013). Sickness absence, marginality, and medically unexplained physical symptoms: a focus-group study of patients’ experiences. Scand J Prim Health Care.

[73] Coole C, Watson PJ, Drummond A (2010). Low back pain patients' experiences of work modifications; a qualitative study. BMC Musculoskelet Disord.

[74] Graff HJ, Deleu NW, Christiansen P (2021). Facilitators of and barriers to return to work after mild traumatic brain injury: A thematic analysis. Neuropsychol Rehabil.

[75] Mårtensson L, Hensing G (2012). Experiences of factors contributing to women's ability to make informed decisions about the process of rehabilitation and return to work: a focus group study. Work.

[76] Porter ME (2008). Value-based health care delivery. Ann Surg.

[77] DeJuisteZorgOpDeJuistePlek. 2022 [cited 2022]. Available from: https://www.dejuistezorgopdejuisteplek.nl/.

[78] VUmc. Patiëntreis als Kerngedachte 2022. Available from: https://www.vumc.nl/nieuws/nieuwsdetail/patientreis-als-kerngedachte.htm.

[79] McCarthy S, O’Raghallaigh P, Woodworth S (2016). An integrated patient journey mapping tool for embedding quality in healthcare service reform. J Decis Syst.

[80] Morrison T, Thomas R, Guitard P (2015). Physicians’ perspectives on cancer survivors’ work integration issues. Can Fam Physician.

