# Supplementary material for: Work-focused healthcare from the perspective of employees living with cardiovascular disease: a patient experience journey mapping study
Source: BMC Public Health. 2023 Sep 11;23:1765. doi: 10.1186/s12889-023-16486-x (PMC10494386; doi:10.1186/s12889-023-16486-x)
Supplement: Supplementary file 1 — Additional file 1: Supplementary material 1. Proportion and position of the participants. Supplementary material 2. The sensitizing booklet. Supplementary material 3. Interview guide. [file 12889_2023_16486_MOESM1_ESM.docx]

Supplementary material

Work-focused healthcare from the perspective of employees living with cardiovascular disease: a patient experience journey mapping study

Content

Supplementary material 1 – Proportion and position of the participants …...2

Supplementary material 2 – The sensitizing booklet..……………………….3

Supplementary material 3 – Interview guide……………………..………….4

**Supplementary material 1 – Proportion and position of the participants**


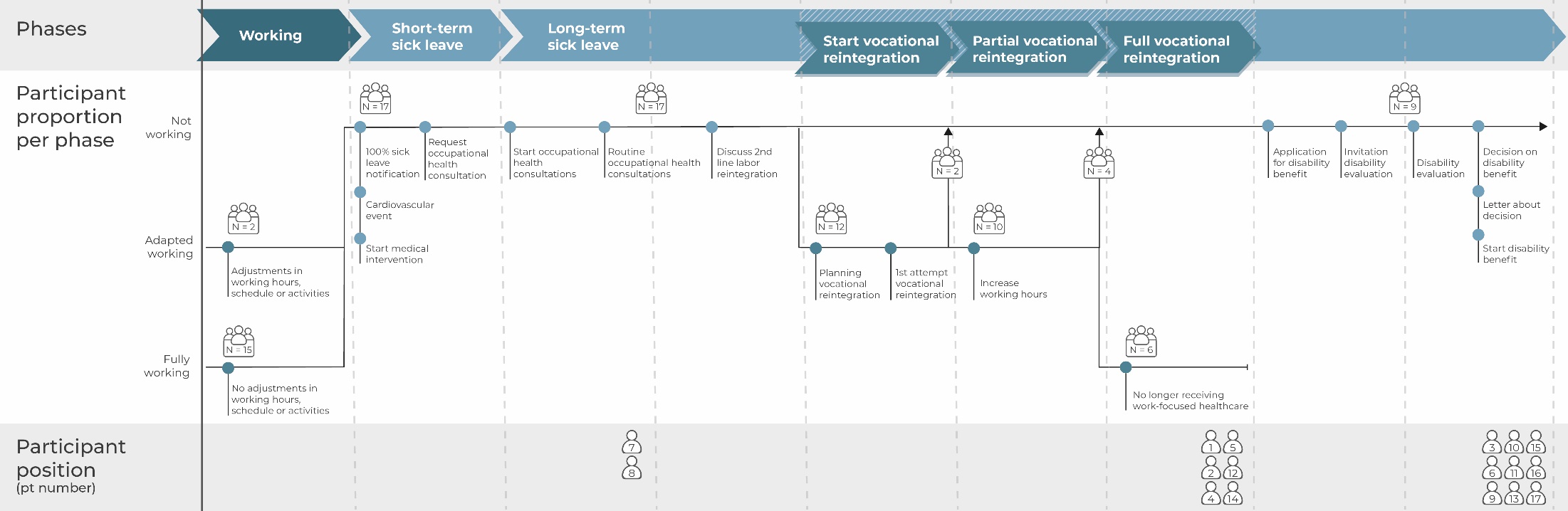


**Supplementary material 2 – The sensitizing booklet**

*Translated from original, Dutch*

1. **PERSONAL INFORMATION**

First of all, we would like to find out more about you, your cardiovascular disease and occupation. Fill in the questions below.

Name: ___________________________________________

Age: _________________ Male/Female

When, and with what form of cardiovascular disease were you diagnosed?

___________________________________________________________________________________________________________________________________________________________________________________________________

Who diagnosed this cardiovascular disease? What caused the diagnosis?

___________________________________________________________________________________________________________________________________________________________________________________________________

What was your occupation at the moment of diagnosis, and what were your work related tasks?

___________________________________________________________________________________________________________________________________________________________________________________________________

Are you currently working in a paid position? What are your work related tasks?

___________________________________________________________________________________________________________________________________________________________________________________________________

1. **STAKEHOLDERS IN YOUR CARE PROCESS**

**Step 1.** Indicate in the table on the next page which stakeholders were involved in the care process for your cardiovascular disease? You can add stakeholders yourself.

*Example*

|  | General practitioner | Disease  Work |  |
| --- | --- | --- | --- |

**Step 2.** Indicate in the table if the stakeholders was involved in the: ‘disease related care process’ and/or ‘work related care process’.

*Example*

|  | General practitioner | Disease  Work |  |
| --- | --- | --- | --- |

**Step 3.** Place the stakeholders involved in your ‘disease related care process’ and ‘work related care process’ in the figure on the page next to the table. You are in the middle. The stakeholders most involved are closer to the middle, and those involved less are further away.

*Example*

**“Disease related care process” “Work related care process”**

General Practitioner

General practitioner

THIS IS YOU

THIS IS YOU

**Step 4.** Mention in a few keywords what you have discussed with those stakeholders involved in your ‘work related care process’.

*Example*

|  | General practitioner | Disease  Work | Fear of job loss,  sick-leave |
| --- | --- | --- | --- |

| STAKEHOLDERS | | …-RELATED  CARE PROCESS | Discussed within  “work related care process” |
| --- | --- | --- | --- |
|  | General Practitioner | Disease  Work |  |
|  | Cardiologist | Disease  Work |  |
|  | Nurse specialist | Disease  Work |  |
|  | Employer | Disease  Work |  |
|  | Colleagues | Disease  Work |  |
|  | Occupational physician | Disease  Work |  |
|  | Insurance physician | Disease  Work |  |
|  | Practice assistant GP care | Disease  Work |  |
|  | Labor expert | Disease  Work |  |
|  |  | Disease  Work |  |
|  |  | Disease  Work |  |
|  |  | Disease  Work |  |
|  |  | Disease  Work |  |
|  |  | Disease  Work |  |
|  |  | Disease  Work |  |

**“DISEASE RELATED CARE PROCESS”**

THIS IS YOU

**“WORK RELATED CARE PROCESS”**

THIS

IS YOU

1. **CHANGES IN WORK DUE TO YOUR CARDIOVASCULAR DISEASE**

**Step 1.** Indicate above the timeline when which changes took plave in your work. Think about: sick leave, adjustments in work, partly working, etc. See the example on the next page.

**Step 2.** Think back about the stakeholders with who you discussed work during your care process (table, discussed within “work-related care process”). Indicate **below** the timeline when you spoke to which stakeholder about these work related topics.

**Step 3.** Indicate for each of these moments whether you experienced this moment as positive (+) of negative (-) during the support for your work problem.

Example step 1

Now

3,5 days/ week working Aug 2020

2 days/ week working June 2020

Sick leave Dec 2019

Full time working

Diagnosis and treatment

Before diagnosis

Example step 2 and 3

Now

Diagnosis and treatment

Before diagnosis

Occupational physician:

Step-by-step return to work (+/-)

Physio:

Focuses on functions needed in my job(+)

Occupational physician:

Optional adjustments in work (-)

Cardiologist:

Taking the time for return to work (+)

1. **COMMUNICATION AND INFORMATION EXCHANGE**

**Step 1.** To your knowledge, which healthcare providers and other involved parties exchange information about your (return to) work process? Think back to your identified stakeholders *(table, stakeholders).*

__________________ with __________________ about __________________

__________________ with __________________ about __________________

__________________ with __________________ about __________________

__________________ with __________________ about __________________

__________________ with __________________ about __________________

**Step 2.** How can the support for (return to) work be improved? How can the stakeholders better meet your wishes and needs? Please, write down all your ideas.

________________________________________________________________________________________________________________________________________________________________________________________________________________________________________________________________________________________________________________________________________________________________________________________________________________________________________________________________________________________________________________________________________________________________________________________________________________________________________________________________________________

____________________________________________________________________________________________________________________________________________________________________

**Supplementary material 3 – Interview guide**

*Translated from original, Dutch*

Theme 1: Diagnosis and disease process

Examples of questions:

- What is your age?
- Which cardiovascular disease do you have?
- When did you receive this diagnosis?
- Where are you now in the treatment process?

Theme 2: Employment, work function and work changes due to CVD

Examples of questions:

- What was your work function before diagnosis?

(work function, how many years, amount of hours a week)

- What type of changes occur in work participation due to the CVD?
- What is your current work situation?

(work function, amount of hours a week, adjustments in work)

Theme 3: Experiences regarding receiving work-focused healthcare

Examples of questions:

- Can you tell us more about with who [this situation] is decided?
- What advice and information have you received from [involved professional]?
- Do you think these were the right moments for support?
- What are your experiences with the usefulness of the advice and information received?
- Did you miss something in terms of support/information from the professionals?
- Have you used any sources of information, such as information?

Theme 4: Communication between different stakeholders

Examples of questions:

- What was your experience regarding communication/cooperation between the professionals involved?
- Of which professionals do you know they communicated at a certain moment in time? Why?
- How can the stakeholders better meet your wishes and needs regarding communication?
