# Supplementary figures and images for: Work-focused healthcare from the perspective of employees living with cardiovascular disease: a patient experience journey mapping study
Source: BMC Public Health. 2023 Sep 11;23:1765. doi: 10.1186/s12889-023-16486-x (PMC10494386; doi:10.1186/s12889-023-16486-x)

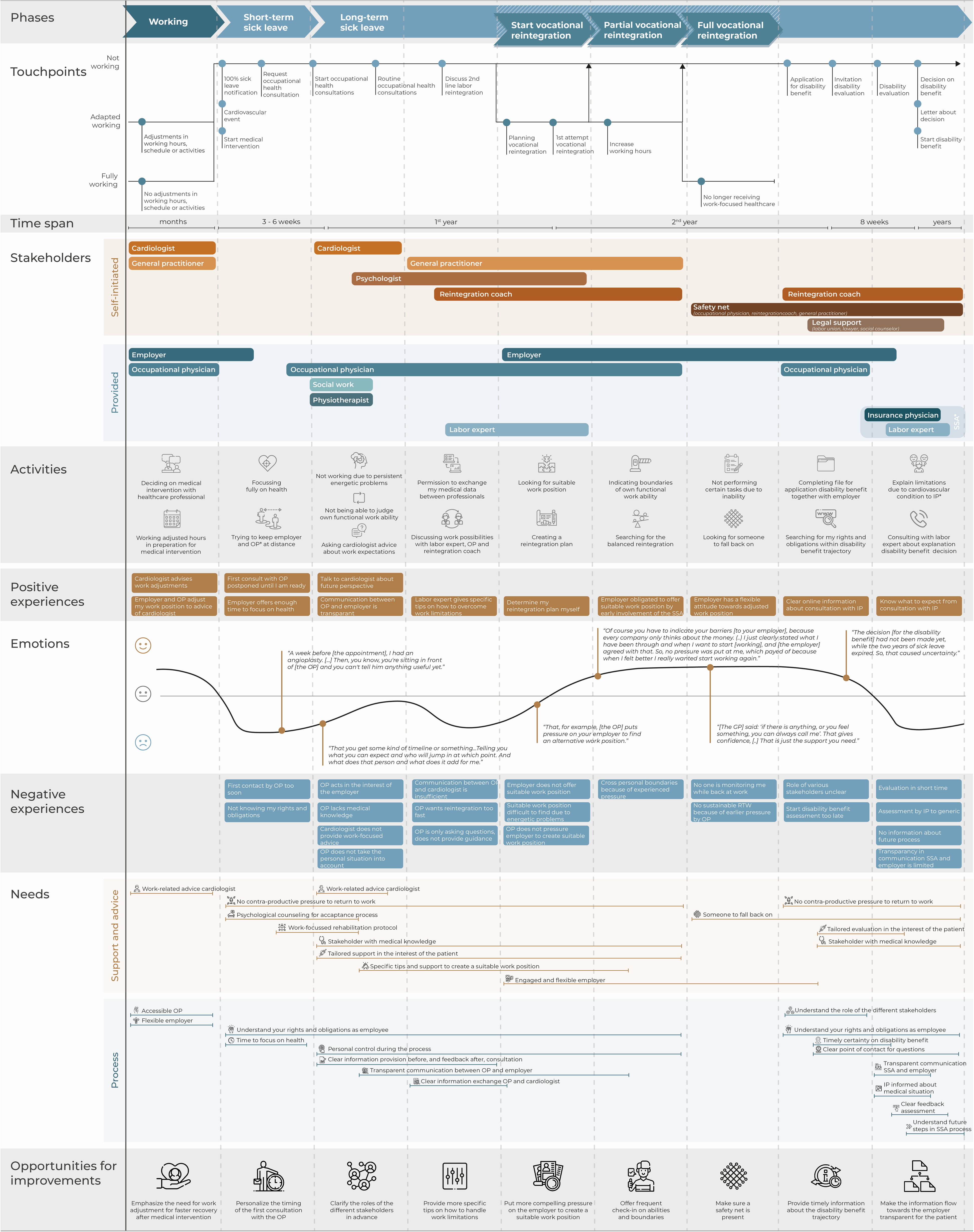

Supplement: Supplementary file 2 — Additional file 2. The work-focused healthcare journey of people living with cardiovascular disease. Legends: Vertical axis show the multiple building blocks this Patient experience journey map exists of. Horizontal axis shows the data of the multiple building blocks over time. IP = Insurance physician, OP = Occupational physician, RTW = return to work, SSA = Social security agency [file 12889_2023_16486_MOESM2_ESM.tif]
